# mGluR5-Antagonist Mediated Reversal of Elevated Stereotyped, Repetitive Behaviors in the VPA Model of Autism

**DOI:** 10.1371/journal.pone.0026077

**Published:** 2011-10-07

**Authors:** Mili V. Mehta, Michael J. Gandal, Steven J. Siegel

**Affiliations:** Translational Neuroscience Program, Department of Psychiatry, University of Pennsylvania, Philadelphia, Pennsylvania, United States of America; Claremont Colleges, United States of America

## Abstract

Autism spectrum disorders (ASD) are highly disabling developmental disorders with a population prevalence of 1–3%. Despite a strong genetic etiology, there are no current therapeutic options that target the core symptoms of ASD. Emerging evidence suggests that dysfunction of glutamatergic signaling, in particular through metabotropic glutamate receptor 5 (mGluR5) receptors, may contribute to phenotypic deficits and may be appropriate targets for pharmacologic intervention. This study assessed the therapeutic potential of 2-methyl-6-phenylethyl-pyrididine (MPEP), an mGluR5-receptor antagonist, on repetitive and anxiety-like behaviors in the valproic acid (VPA) mouse model of autism. Mice were exposed prenatally on day E13 to VPA and assessed for repetitive self-grooming and marble burying behaviors as adults. Anxiety-like behavior and locomotor activity were measured in an open-field. VPA-exposed mice displayed increased repetitive and anxiety-like behaviors, consistent with previously published results. Across both marble burying and self-grooming assays, MPEP significantly reduced repetitive behaviors in VPA-treated mice, but had no effect on locomotor activity. These results are consistent with emerging preclinical literature that mGluR5-antagonists may have therapeutic efficacy for core symptoms of autism.

## Introduction

Autism spectrum disorders (ASD) are neurodevelopmental disorders characterized by repetitive and restricted patterns of behavior, reduced social interactions, and impairments in language function. Recent estimates suggest that autism has a population prevalence of 1–3%, with symptom onset beginning within the first three years of life [1], [2], [3]. Although not considered diagnostic symptoms, autism is characterized by a number of neurological and psychiatric comorbidities, such as anxiety. Of the core domains, repetitive behaviors are of particular interest because they are the strongest predictor that an early diagnosis of ASD will persist throughout the lifetime [4]. These behaviors, with a similarity to symptoms of obsessive-compulsive disorder (OCD), include stereotypic movements, repetitive play, inflexible routines, and a ritualistic insistence on sameness [5]. When such behaviors are interrupted, a child may protest or exhibit anxiety or aggression [5]. The severity of many of these disorders, combined with the high prevalence rates, highlights the need for the development of biological interventions. However, second generation antipsychotics, such as risperidone, are the only FDA-approved class of medications for the treatment of ASD. There is some evidence that risperidone may improve repetitive, restricted interests and behaviors in subjects with autism [6], [7], one of the core symptomatic domains, although results are mixed [8], [9]. Treatment with risperidone is generally limited to patients who display certain associated maladaptive behaviors, such as irritability, aggression, and self-injury [10], [11], as the drug has a significant side-effect profile, including weight gain and metabolic syndrome, which complicate its therapeutic potential [12]. As such, there is a strong need to develop novel therapeutic approaches that target core deficits (and pathophysiology) of autism, while limiting adverse effects.

Investigation of novel therapeutics requires a robust preclinical paradigm with a validated animal model that displays phenotypes analogous to the core symptoms of autism. We recently demonstrated that a single prenatal (embryonic day 13) exposure to the anticonvulsant valproic acid (VPA), which is associated with a 7–10 fold increased relative risk for ASD in humans, recapitulates selective behavioral and electrophysiological deficits in mice analogous to those seen in the clinical population [13], [14], [15]. In particular, VPA-exposed mice demonstrated increased repetitive self-grooming behaviors, decreased social preference, and reduced emission of ultrasonic vocalizations thought to reflect communicative dysfunction [15]. Similar findings have been reported in rats exposed to VPA *in utero*, bolstering the validity of this model [16].

Current theories of ASD pathogenesis attribute symptoms to the disrupted balance of excitatory/inhibitory signaling during critical developmental periods [17], [18]. In accordance with this theory, prenatal VPA exposure causes lasting changes in neural circuitry leading to heighted excitation and reduced inhibition. Such disruptions may be restored by targeted pharmacotherapeutic agents that improve excitatory-inhibitory balance. Glutamate is the main excitatory neurotransmitter in the brain and utilizes two different receptor types, ionotropic and metabotropic G-protein coupled receptors. Emerging evidence implicates disrupted glutamatergic signaling in autism and related disorders, in particular signaling that involves the metabotropic glutamate receptor 5 (mGluR5) receptor [19], [20]. As such, recent preclinical work has investigated the therapeutic potential of 2-methyl-6-phenylethyl-pyrididine Specifically, MPEP reduced stereotypies in the BTBR mouse model of autism [21] and reversed ASD-like endophenotypes in the VPA mouse model [15].

This study sought to extend these previous findings by investigating the potential therapeutic effects of MPEP on repetitive behaviors in the VPA mouse model of ASD. We chose to start by measuring repetitive self-grooming and marble burying behaviors, two motor stereotypies that have been previously investigated in ASD mouse models, as the more complex insistence on sameness has yet to be fully investigated with pharmacology in mice. Finally, we employed open field and locomotor activity paradigms to assess potentially confounding sedative effects of MPEP and the possibility that the efficacy of MPEP may be due to its anxiolytic properties.

## Materials and Methods

### Animals

C57BL/6Hsd (B6) mice were obtained at 7–8 weeks of age from Harlan (Indianapolis, Indiana) and were mated, with pregnancy confirmed by the presence of a vaginal plug on embryonic day 0 (E0). On E13, pregnant females received a single subcutaneous injection of 600 mg/kg VPA (Tocris BioScience, Ellisville, Missouri) dissolved in saline (SAL). Control females received an equal volume of SAL only. There were 56 pups delivered from 10 VPA-treated dams, and 34 pups from 6 SAL-treated females. Of those, 24 pups from VPA-treated dams and 24 pups from SAL-treated dams were used in this study. Day of birth was recorded as P0. Animals were maintained in a standard 12-hour light/dark cycle with free access to food and water. The same 24 mice were used in each assay per group. Half of each group received an injection of MPEP prior to each assay, while the rest received an injection of vehicle. Behavioral testing was performed in the following order: self-grooming, locomotor activity/open field, and marble burying. There were on average 16 days between self-grooming and locomotor activity testing sessions and 7 days between LMA and marble burying testing. All protocols were approved by the University of Pennsylvania Institutional Animal Care and Use Committees and were conducted in accordance with National Institutes of Health guidelines.

### Self-Grooming Assay

Mice were scored for spontaneous grooming time as a measure of repetitive behavior from P42 to P50, according to our published methods [15]. Each mouse was placed in a clean empty plastic cage in white light (400 lux) for a 10-minute acclimation period. Bedding was not used in order to prevent digging, a potentially competitive repetitive behavior. Following acclimation, mice were scored with a stopwatch for cumulative time spent self-grooming during a 10-minute testing period. Between each testing period, the cage was cleaned with 70% ethanol, left to dry for 5 minutes, and wiped down with a clean paper towel.

### Open Field and Locomotor Testing

From P56 to P64, assessment of anxiety-like behavior and locomotor activity was performed in a (50×35×40 cm) open field as published, on average 16 days after testing in the self-grooming assay [15]. Briefly, mice were placed individually in the periphery of the open field boxes covered with 2 cm of bedding under white light (400 lux) for 30 minutes. Video was digitally recorded and movements were traced with TopScan software (CleverSys, Reston, VA). A peripheral (11 cm width) and a central zone (30 cm×11 cm) were defined and the movements of each mouse were traced. Anxiety-like behavior was assessed for each mouse by the number of entries into the center zone, as well as the time spent in this region during the first ten minutes of the testing session. Locomotor activity was recorded as total distance traveled (mm per minute) for each mouse and significance was assessed with a group x drug x minute repeated-measures ANOVA.

### Marble Burying Assay

Mice were assessed in the marble-burying assay from P65 to P71, on average 7 days after open field testing. Mice were allowed to acclimate individually in a (50×35×40 cm) cage filled with 4 cm of fresh bedding for 15 minutes in white light (400 lux). To minimize neophobia and novelty-induced anxiety, mice had been previously exposed to the testing arena during locomotor and open field testing. Following acclimation, mice were removed from the cage, and 20 black marbles were placed equidistant in a 4×5 arrangement. Mice were then returned to the same cage for 30 minutes. Following the 30-minute testing period, the number of marbles that were more than two-thirds covered with bedding were counted by a single blinded experimenter. After each testing period, the cage was cleaned with 70% ethanol, left to dry for 5 minutes, wiped down with a clean paper towel, and fresh bedding was then added.

### Pharmacology

2-methyl-6-phenylethynyl-pyridine (MPEP) was obtained from Sigma and dissolved in 0.9% saline at 20 mg/kg prior to testing session. Dosages were based on previous publications demonstrating efficacy in mice [21]. Intraperitoneal injections of saline vehicle or drug were given 10 minutes before onset of behavioral assays. Previous work has demonstrated that the maximal occupancy of MPEP at mGluR5 receptors in mouse forebrain occurs 10 minutes after i.p. injection [22].

### Statistical Methods

For each behavior, statistical significance was assessed with a group (SAL, VPA) x drug (VEHICLE, MPEP) ANOVA using GraphPad Prism software (v5, La Jolla, CA). Where appropriate, Bonferroni post-tests were calculated to determine significance between individual groups using the GraphPad Prism post-test calculator (http://www.graphpad.com/quickcalcs/posttest1.cfm). For locomotor activity, statistical significance was assessed with a group (SAL, VPA) x drug (VEHICLE, MPEP) x minute (1–30) repeated-measures ANOVA using Statistica software (v6, Tulsa, OK).

## Results

### Repetitive Self-Grooming Behavior

**Figure 1:**
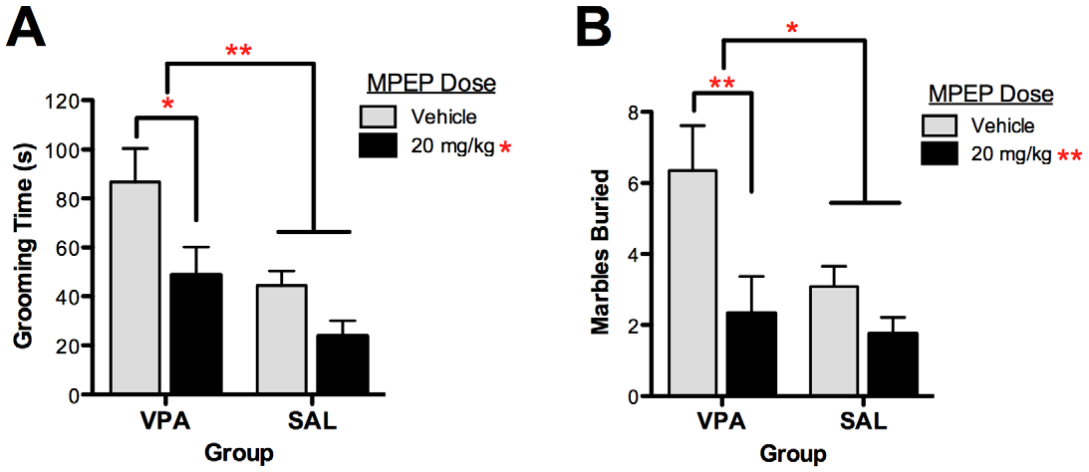
The mGluR5-receptor antagonist MPEP attenuates elevated repetitive behaviors in mice exposed to valproic acid (VPA) *in utero.* (**A**) Repetitive self-grooming was measured over 10 minutes in mice exposed to prenatal saline (SAL) or VPA. (**B**) Repetitive marble burying behavior was measured for both groups after a 30 minute testing session. Across both assays, VPA-exposed mice demonstrated elevated stereotyped, repetitive behaviors that were significantly reduced by MPEP. Figures show mean ± S.E.M., (*p<0.05, **p<0.01).

The self-grooming assay was used to test the effects of MPEP on repetitive behavior in mice exposed prenatally to valproic acid (VPA) and saline (SAL). Self-grooming activity has been widely employed as an index of repetitive behavior relevant to the core symptom domain in several other preclinical studies of autism [20], [21], [23], [24]. A main effect of group (F(1,47) = 9.361, P<0.004) indicated that VPA-exposed mice groomed themselves significantly more than SAL-exposed mice over the 10-minute testing session, consistent with previous results (**Fig 1a**) [15]. In addition, treatment with MPEP significantly reduced self-grooming behavior across both groups (F(1,47) = 7.10, P = 0.011). There was no significant group by drug interaction. Drug effects were driven by the VPA group, which was significantly different between drug conditions (*P*<0.04, Bonferroni post-test), whereas the SAL group did not differ between vehicle and MPEP treatments (*P*>0.05, Bonferroni post-test). In addition, the grooming times of the MPEP treated VPA-exposed mice were comparable to those of the vehicle treated SAL-exposed mice (*P*>0.05, Bonferroni post-test). These results demonstrate that MPEP normalizes the increased repetitive behaviors following *in utero* VPA exposure.

### Marble Burying Assay

In addition, we assessed the effect of MPEP on marble burying activity in VPA- and SAL-exposed mice. This assay has been employed as a measure of anxiety-like and repetitive OCD-like behaviors in mice, although it has been debated which domain it more closely evaluates [25]. VPA-exposed mice buried significantly more marbles than did SAL-exposed mice, consistent with an increase in stereotyped, perseverative behaviors (F(1,46) = 4.25, *P*<0.05; **Fig 1b**). When treated with MPEP, the number of marbles buried was significantly reduced across both groups (F(1,46) = 8.20, *P* = 0.006). There was no significant group by drug interaction. Again, although there was a main effect of drug across both groups, post-tests revealed that this effect was driven by the VPA group. Marble burying was significantly reduced in VPA-exposed mice treated with MPEP compared to vehicle (*P*<0.01, Bonferroni post-test). However, in the SAL-exposed group, there was no difference between vehicle and MPEP conditions (*P*>0.05, Bonferroni post-test). Finally, the number of marbles buried by MPEP treated VPA-exposed mice was comparable to those buried by vehicle treated SAL-exposed mice (*P*>0.05, Bonferroni post-test). These results further demonstrate that MPEP is effective at reducing the increase in stereotyped, repetitive marble burying caused by prenatal VPA exposure.

### Open Field Testing

**Figure 2:**
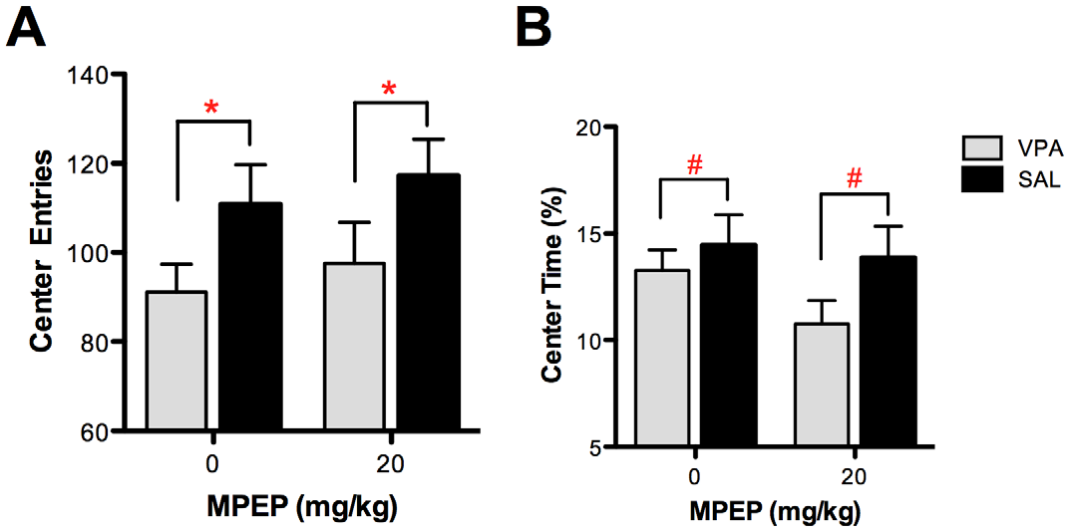
The effect of MPEP was assessed on anxiety-like behavior in VPA- and SAL-exposed mice using an open-field paradigm. Consistent with elevated anxiety, VPA-exposed mice demonstrated significantly fewer (**A**) center entries and qualitatively reduced (**B**) center time. Across groups, there was no effect of MPEP on either of these measures. Figures show mean ± S.E.M., (#*p*<0.1, *p<0.05).

To determine if the effect of MPEP was mediated simply by reducing anxiety-like behavior in the VPA group, we next assessed this behavioral domain with an open-field paradigm. Consistent with previous reports of increased anxiety-like behavior in this model [15], [26], VPA-exposed mice exhibited significantly fewer center entries than SAL-exposed mice (F(1,43) = 5.65, P = 0.02; **Fig 2a**), although center time did not reach statistical significance between groups (F(1,43) = 2.85, P = 0.09; **Fig 2b**). There was no effect of MPEP on the number of center entries (F(1,43) = 0.61, P* = *0.44) or percentage of time spent in center (F(1,43) = 1.46, P = 0.23). These results indicate that the positive effect of MPEP on stereotyped, perseverative behaviors in the VPA model is not likely mediated by reducing concomitant anxiety-like behavior.

### Locomotor Activity

**Figure 3:**
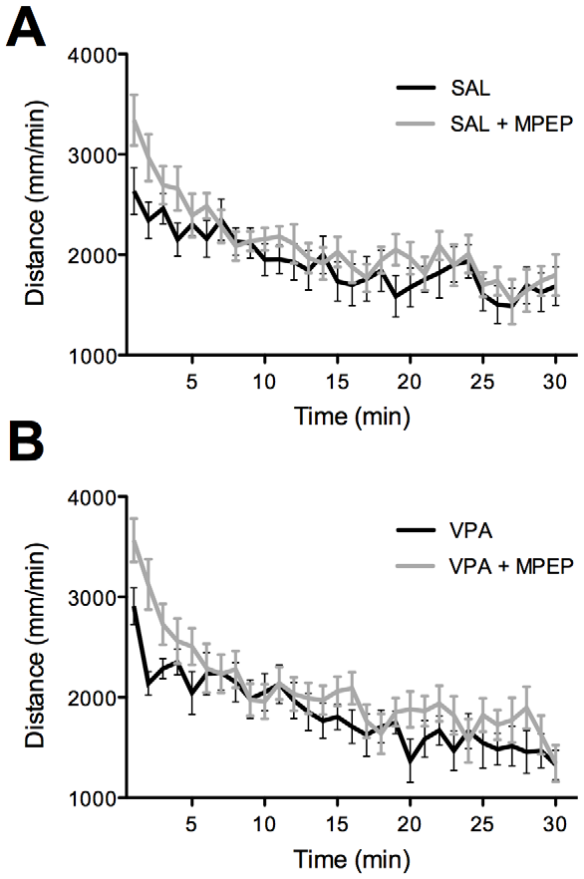
The effect of MPEP on spontaneous locomotor activity (LMA) was assessed across groups as sedative effects could potentially confound findings in other behavioral assays. As previously reported, there was no significant difference in locomotor activity between groups. Likewise, MPEP did not significantly alter locomotor activity in (**A**) SAL- or (**B**) VPA-exposed mice over the 30 minute testing session. MPEP caused a qualitative increase in LMA across groups, but only for the first 5 minutes following drug injection, further supporting a lack of sedative effects.

Finally, a sedating drug effect could potentially confound observed results in marble burying and self-grooming assays. To address this, the effect of MPEP was assessed on locomotor activity (LMA) in VPA- and SAL-exposed mice over a 30-minute testing session. Significance was assessed with a group x drug x minute repeated measures ANOVA. Consistent with our previous findings, VPA-treated mice showed no difference in baseline LMA compared with SAL controls (F(1,43) = 0.24, P = 0.63) [15]. Likewise, there was no significant effect of MPEP on LMA (F(1,43) = 3.17, P = 0.09; **Fig 3**), nor was there a significant group by drug interaction (F(1,43) = 0.04, P = 0.84). Although not significant, there was a qualitative trend towards increased locomotor activity with MPEP, mediated mostly by the first 5-minutes of testing, after which drug conditions were indistinguishable among groups. This suggests that overly sedating effects of MPEP did not confound the repetitive and anxiety-like behavioral results reported above.

## Discussion

The present study investigated the effects of the mGluR5 antagonist MPEP on measures of anxiety and stereotyped, repetitive behaviors in a mouse model relevant to autism. We demonstrate that MPEP displays efficacy in reducing repetitive self-grooming and marble burying behaviors in mice exposed *in utero* to valproic acid. There was no effect of MPEP on anxiety-like behavior in an open field paradigm, nor were our results confounded by overly sedating pharmacological effects.

### MPEP: Efficacy and Mechanism of Action

These findings complement previous work demonstrating positive effects of mGluR5 antagonists on phenotypic deficits relevant to the core symptoms of autism in preclinical studies. Of note, Silverman and colleagues demonstrated a similar reduction in repetitive behaviors following MPEP administration in BTBR mice, an inbred genetic model relevant to ASD [21]. In the VPA mouse model, we previously showed that MPEP reverses autism-like endophenotypes, including deficits in prepulse inhibition (PPI) and reduced auditory-evoked gamma frequency synchrony [15]. Likewise, a number of studies have shown that mGluR5 antagonists are effective in reversing neural deficits in preclinical models of Fragile X Syndrome (FXS), which shares a high degree of overlap with autism. For example, MPEP has been demonstrated to reverse PPI and dendritic spine abnormalities in *Fmrp1* knockout mice, as well as restore social courtship behavior defects in a *Drosophila* model of the disease [27], [28]. Demonstrating positive therapeutic effects of this drug across several distinct preclinical models bolsters the robustness of these findings and the generalizability of the therapeutic potential of mGluR5 antagonists for the treatment of autism. MPEP has also displayed analgesic and anxiolytic actions in other models of disease, properties that can also benefit patients with autism who often exhibit increased sensitivity to sensory stimuli, as well as anxious and self-injurious behaviors [21], [29], [30], [31].

The effectiveness of MPEP in reducing stereotypic behaviors fits with emerging evidence that glutamatergic dysfunction may contribute to core deficits in autism [19], [20], [32]. Current theories of disease pathogenesis implicate a disrupted balance of excitation and inhibition, a balance that may be restored by blocking mGluR5 receptors [15], [17]. Indeed, previous work has shown that mice exposed *in utero* to VPA have elevated glutamatergic signaling and hyperexcitable local circuitry [33], [34], which likely contributes to the abnormal phenotypes seen in this model. Increased excitatory signaling could be attributed to reduced inhibition, as previous work has also demonstrated a loss of parvalbumin-expressing interneurons following *in utero* VPA exposure [18]. Collectively, these findings suggest that attenuating elevated glutamatergic signaling, with drugs like MPEP, could ameliorate behavioral abnormalities in the VPA model, as we have shown in this study. Such restoration may reflect a reduction of NMDAR-mediated currents, as mGluR5 receptors are known to reciprocally modulate NMDAR signaling [35]. Finally, there is growing evidence that glutamate-modulating agents may be efficacious in treatment-resistant OCD, further demonstrating the role of glutamatergic systems in stereotypic behaviors [36].

### MPEP and Anxiety

It has been difficult to separate repetitive OCD-like and anxiety-like behaviors, particularly in preclinical studies. For example, OCD is considered an anxiety disorder, and there is frequently significant clinical overlap between OCD and generalized anxiety disorder [37]. Several mouse behavioral paradigms have been proposed to assess the repetitive, restricted behaviors and insistence on sameness, which comprise the third core symptomatic domain of autism [24], [38], [39], [40], [41], [42]. T-maze and water-maze reversal learning paradigms have been proposed to reflect cognitive inflexibility, whereas marble burying and self-grooming have been widely employed as measures of motor stereotypies. However, both marble burying and self-grooming assays have also been shown to be sensitive to anxiety [43], [44], although there is some controversy as to which behavior each task is most sensitive [25], [45].

Our data demonstrate that acute administration of MPEP is sufficient to reduce elevated self-grooming and marble burying behaviors in VPA-exposed mice but has no effect on open-field activity, a traditional test for anxiety-like behaviors. However, our results do not definitively demonstrate that these effects are independent of the anxiolytic effects of MPEP. Several additional tests of anxiety-like behaviors, such as light-dark exploration and open arm entries on an elevated plus-maze, would be required to conclusively rule out the possibility that the effects of MPEP are mediated by anxiolysis. Nevertheless, reducing stereotyped, repetitive behaviors as well as anxiety-like behaviors would both be beneficial for autism, given that individuals with ASD frequently have comorbid anxiety disorders [3].

### Limitations and Future Directions

In conclusion, our results support the therapeutic potential of MPEP for the treatment of repetitive, stereotyped behaviors relevant to patients with autism. This study provides mechanistic insight into the pathophysiology of OCD-like repetitive behaviors relevant to autism. However, it is unclear whether MPEP, itself, would be well tolerated in the clinical population and would thus be a good drug candidate for clinical trials.

One limitation of our study is the generalizability of the VPA mouse model to the ASD population. Although many of the behavioral and neuropathological deficits following prenatal VPA exposure mimic those seen in ASD patients, the majority of such patients were not exposed to this drug *in utero*. However, the efficacy of MPEP on repetitive behaviors replicated across multiple preclinical ASD models of autism strengthens the predictive validity of these findings.

Whereas our study did not find a significant effect of MPEP on anxiety-like behavior across VPA- and SAL-treated mice, other studies have reported anxiolytic effects of mGluR5 antagonists in rodent models of anxiety using various behavioral paradigms [46], [47]. This discrepancy could reflect the use of lower doses, acute (versus chronic) administration, different behavioral paradigms, or the lack of a fear/stress-inducing behavioral test such as one involving foot-shocks [47], [48], [49]. However, the observation that MPEP was effective in reducing repetitive behaviors acutely in the VPA model suggests that its efficacy towards these behaviors may be more selective and/or more immediate than that for anxiety.

Future studies will investigate the therapeutic potential of MPEP on additional behaviors relevant to the core symptoms of autism (e.g., social deficits). We chose to begin with stereotyped behaviors given that previous work failed to find an effect of MPEP on sociability in the BTBR model [21]. Likewise, future studies will also attempt to further reconcile the distinct pharmacologic profile of MPEP on anxiety versus OCD-like behaviors by employing a chronic dosing strategy, as well as by utilizing additional measures of anxiety and repetitive behavior in rodents.
